# Knowledge, attitudes and practices related to *Taenia solium* cysticercosis and taeniasis in Tanzania

**DOI:** 10.1186/s12879-022-07408-0

**Published:** 2022-06-13

**Authors:** Chacha Nyangi, Dominik Stelzle, Ernatus M. Mkupasi, Helena A. Ngowi, Ayubu J. Churi, Veronika Schmidt, Christopher Mahonge, Andrea S. Winkler

**Affiliations:** 1 Department of Veterinary Medicine and Public Health, Sokoine University of Agriculture, Morogoro, Tanzania; 2 Department of Applied Sciences, Mbeya University of Science and Technology, Mbeya, Tanzania; 3 Center for Global Health, Department of Neurology, Technical University of Munich (TUM), Munich, Germany; 4 Chair of Epidemiology, Department of Sports and Health Sciences, Technical University Munich (TUM), Munich, Germany; 5 Centre for Information and Communication On Technology (CICT), Sokoine University of Agriculture, Morogoro, Tanzania; 6 Centre for Global Health, Institute of Health and Society, University of Oslo, Oslo, Norway; 7 Department of Policy, Planning and Management, Sokoine University of Agriculture, Morogoro, Tanzania

**Keywords:** Knowledge, Attitude, Practices, Smallholder pig farming, Taenia solium, Cysticercosis, Taeniasis, Epilepsy, Neurocysticercosis, Cross-sectional study

## Abstract

**Background:**

*Taenia solium* cysticercosis/taeniasis (TSCT) is reported to be endemic in pig producing areas around the world, causing significant disease burden and economic losses.

**Methods:**

This cross-sectional study aimed at assessing Knowledge, Attitudes and Practices (KAP) regarding TSCT in four districts, namely Mbulu, Mpwapwa, Mbinga, and Rungwe in Tanzania. Data on KAP were collected through questionnaire-based interviews and household infrastructure observations.

**Results:**

Knowledge about porcine cysticercosis was good, particularly among pig keepers across the districts. Many participants had heard about the pork tapeworm (*T. solium* taeniasis), and the knowledge about signs/symptoms and treatment was fair, but the means of transmission and prevention measures were often unknown. Whilst most participants were familiar with epilepsy, no one knew anything about human cysticercosis and the link between cysticercosis and epileptic seizures. A similar trend is reflected through the attitudes toward the low risk perception of cysticercosis infection. Not surprisingly, the risk perception of the infection with the pork tapeworm was low too. Many participants reported not washing their hands before eating or after using the toilet which highlights potential risks for the development of human cysticercosis. Albeit nearly every participant reported using the toilet always, household observations revealed that toilets were either lacking or had no complete walls. Generally, household observations revealed a discrepancy between questionnaire answers on the one hand and the availability of toilet and handwashing facilities and the confinement of pigs on the other hand.

**Conclusion:**

This study demonstrates knowledge gaps and adverse practices which may hinder and/or slow down the control/elimination of *T. solium* in endemic countries. The study results are also useful for appropriate designing of TSCT health interventions that need to be planned carefully, taking into account the local context and designing TSCT in partnership with the local communities from the beginning to the end applying a One Health approach to allow the possible sustained and best impacts.

**Graphical Abstract:**

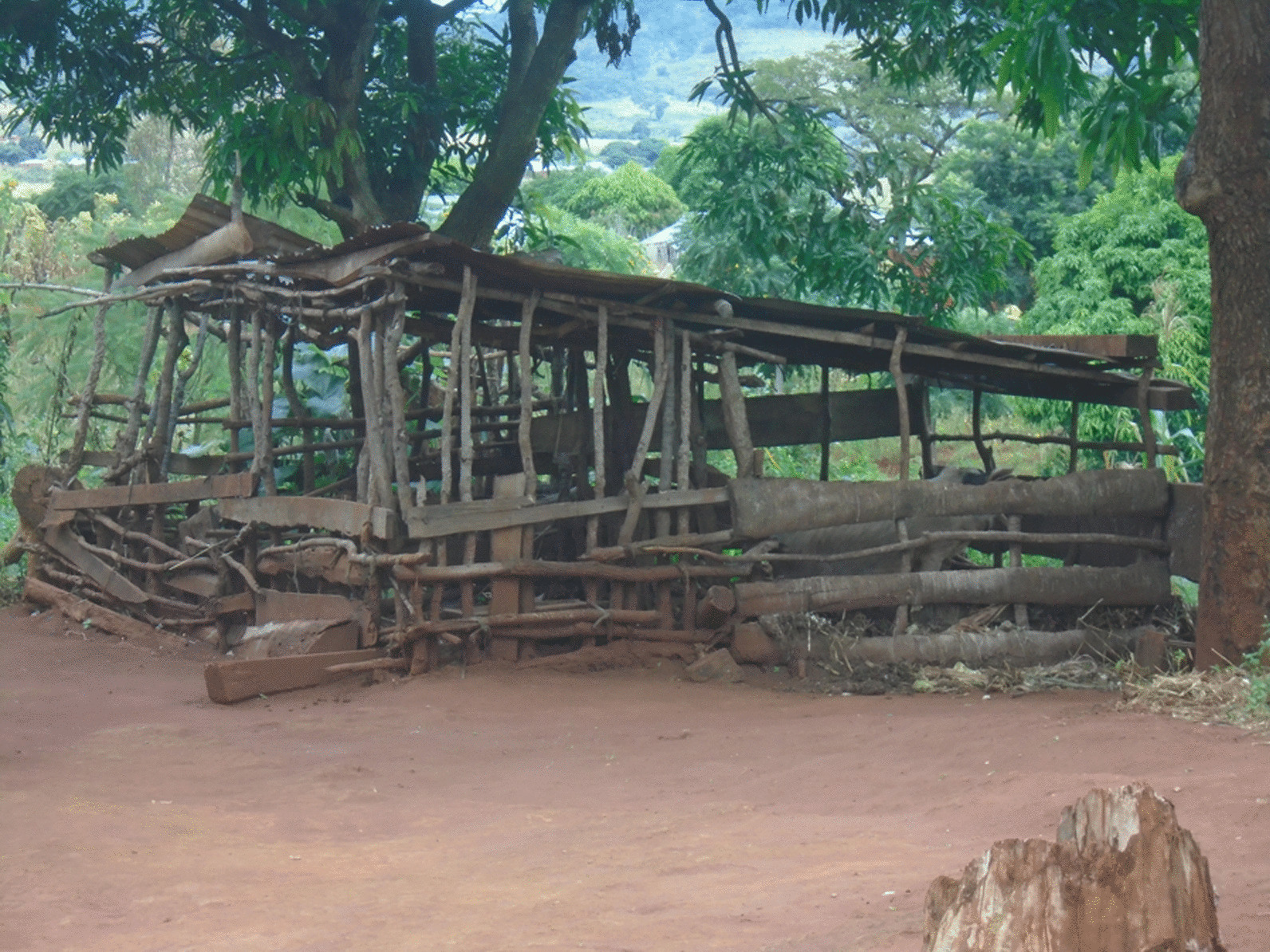

**Supplementary Information:**

The online version contains supplementary material available at 10.1186/s12879-022-07408-0.

## Background

*Taenia solium* cysticercosis/taeniasis (TSCT) is a parasitic zoonotic disease with severe human/veterinary public health and economic impacts [1]. TSCT is the global number one parasitic foodborne disease as ranked by the World Health Organisation (WHO) [2]. The disease occurs more frequently in countries with poor socio-economic development [3, 4]. Areas with low standards of personal hygiene, poor environmental sanitation, poor pig management, inadequate meat inspection, and limited knowledge of TSCT [5, 6] are the ones severely affected by the disease.

Humans, the final host, harbour the adult tapeworm in their intestines, this condition is referred to as taeniasis; human faeces release infective eggs into the environment [7]. Pigs, the principal intermediate host, get infected through ingestion of eggs from contaminated feed (fruits e.g. pawpaw and mangoes, vegetables, green leaves, leftovers, maize bran, etc.) water, or through direct ingestion of human faeces. The eggs in the intestine release an embryo (an oncosphere), which migrates to the striated muscles and other tissues where it matures into the larval stage (metacestode), commonly known as cysticercus (singular) or cysticerci (plural) [3]. Humans acquire taeniasis through consuming raw or undercooked infected pork. Cysticerci develop in the small intestine into adult tapeworms leading to taeniasis. After about two months, they mature and start producing eggs, which are passed with faeces isolated as well as inside the gravid proglottids, thus the lifecycle of the parasite between the human and pig hosts is completed [8]. Humans can also act as intermediate hosts and develop cysticercosis after ingestion of eggs from food or water contaminated with human faeces (faecal-oral transmission) or through autoinfection [7]. The development of larvae in the central nervous system tissue, which includes the brain and the spinal cord, leads to neurocysticercosis (NCC). NCC may result in severe or even fatal neurological conditions, which are often manifested through epileptic seizures or epilepsy, severe chronic headaches, and/or focal neurological deficits [1].

Despite the efforts of controlling/eliminate TSCT, the disease is still endemic in many pig-raising regions in Tanzania and around the world, particularly in sub-Saharan Africa, Southeast Asia and Latin America. Several studies using various diagnostic methods in pig keeping areas of Tanzania reported estimates of the prevalence of porcine cysticercosis (PCC) of between 11.7 and 13% based on lingual examination [9, 10], and 32% based on antigen-based enzyme-linked immunosorbent assay (Ag-ELISA) [9]. The estimates of the prevalence of human cysticercosis (HCC) were between 16.7% using Ag-ELISA and 45.3% using an antibody (Ab)-ELISA [5]. In two neuroimaging studies from rural northern Tanzania, the estimates of the prevalence of NCC among people with epilepsy ranged from 4 to 18% [11, 12], but the overall estimates go even up to 30% prevalence of NCC in people with epilepsy in endemic areas [13, 14]. The societal costs in communities where TSCT is endemic are extremely high. In Tanzania, economic loss was estimated at around 5 million USD per year due to NCC-related epilepsy, and nearly 3 million USD due to PCC for the year 2012 [15]. Poor knowledge regarding risk factors, transmission, signs/symptoms in humans and animals, prevention, and treatment may cause the disease to remain endemic in most regions.

Community knowledge, attitudes, and practices (KAPs) are vital in establishing successful control/elimination strategies for various infections [16]. Adequate knowledge motivates people to adapt control strategies such as the need for treatment of tapeworm infection or improved sanitation, hygiene, and pig-rearing practices, which, in turn, may also help in reducing faecal-oral transmission of various other diseases. KAP survey data can help to recognize gaps in knowledge, cultural beliefs, or behavioural patterns that may facilitate understanding and action, as well as pose problems or create barriers against the disease (TSCT) control/elimination efforts [16]. However, information on KAPs about TSCT in many African countries is limited [6, 17–21]. Accordingly, the current study aimed to evaluate the KAPs of TSCT in endemic districts namely, Mbulu, Mpwapwa, Mbinga, and Rungwe in Tanzania, The results of the current study will also serve as the basis for the development of a contextualised TSCT health education package that will be implemented locally for TSCT control/elimination purposes.

## Methodology

### Study area

**Fig. 1:**
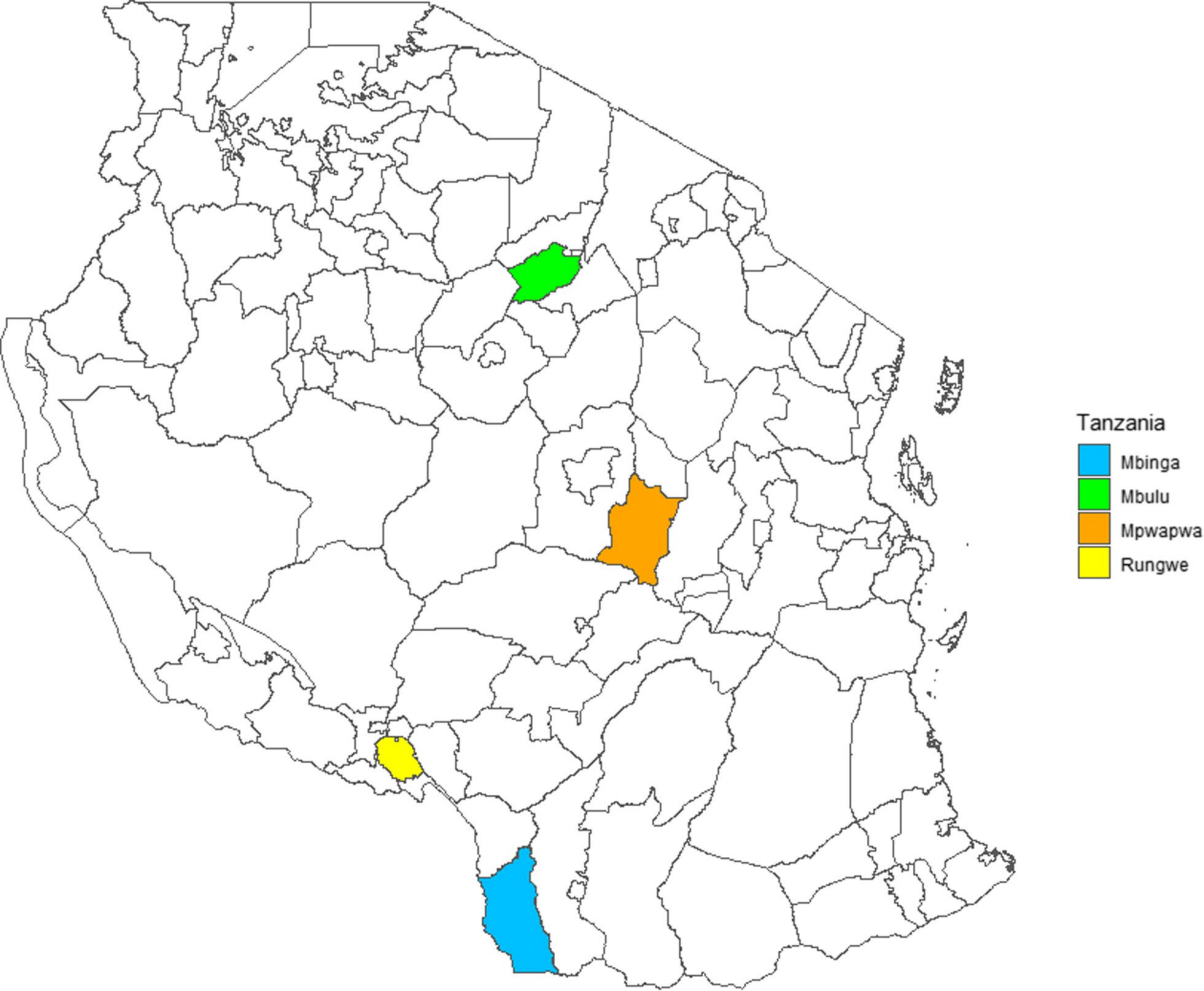
Map of Tanzania showing the study districts. The map was created using the R package “sf” version 0.8–0. The shapefile was extracted from the GADM database (www.gadm.org), version 2.5, July 2015

This cross-sectional study was conducted between August 2018 and June 2019 in four selected districts: three districts namely Mbulu (Manyara Region), Mpwapwa (Dodoma Region), and Mbinga (Ruvuma Region) (Fig. 1) were purposely selected for being PCC endemic areas and popular in small-scale pig rearing [6, 18, 20, 22–26]. The fourth district, Rungwe (Mbeya Region) (Fig. 1), was selected for comparison purposes because the endemicity of *T. solium* is unknown. Nonetheless, the district is popular for small-scale pig rearing and borders neighbouring districts known to be endemic to PCC.

### Study design

This cross-sectional study consisted of a questionnaire-based survey and direct household infrastructure observation conducted by the main researcher in one village from each of the eight purposely selected wards in the four aforementioned districts (two wards per district). In each village, 60 households were randomly selected, 30 pig keepers and 30 non-pig keepers. A list of all households of pig farmers and non-pig farmers is kept at village level in the office of the village chairperson. The households were stratified according to sub-villages from which they were selected randomly. For pig keepers as they were few, every 2nd name was chosen and for non-pig keepers as they were many every 10th name was chosen. The involvement of pig keepers and non-pig keepers was important for the development of a health education package. In each of the selected households, an interview was conducted with the head of the household. In case the head was absent, another adult household member was interviewed. In addition, a clear colour picture of an adult tapeworm and another of pork infested with cysticerci were shown to the respondents during the interview to ensure that correct information was obtained when referring to a “tapeworm” and “cysticerci” and to reduce the possibility of confusing these with other parasites (Additional file 1). There is a specific name for pork tapeworm (“mnyoo tegu wa nguruwe”) in Kiswahili, the local language, which refers to pork tapeworm only, and not to tapeworm overall. This is why when asking” Have you heard about the intestinal tapeworm called *T. solium*?” in Kiswahili and pointing at the picture of the pork tapeworm at the same time, we could be relatively sure that the answers of our participants referred to *T. solium* and not *T. saginata*.

### Sample size determination

The sample size was computed using the formula by Fisher [27] assuming a significance level of alpha = 0.05 and accounting for four different study areas. Since the level of knowledge, attitude, and practices was not known, the overall frequency of 50% was assumed which yielded the minimum sample size required for this study (n = 384). We added 25% to account for a non-response and yielded a total number of 480 households.

### Questionnaire survey

The questionnaire was designed based on the WHO guide for developing KAP surveys [16]. We also consulted previous studies on KAP in sub-Saharan Africa [17–21] which inspired us to develop our questions. We phrased our questions according to the local circumstances and piloted the questionnaire as described below.

The questionnaire was administered face-to-face by a team of three investigators (CN, CM, DM; the last two are in the acknowledgement section) and administered separately to each of the selected household members. The questionnaire contained 45 questions altogether. The questionnaire is attached in the supplementary material (Additional file 1: Questionnaire). The general part of the questionnaire contained 7 questions. Knowledge about TSCT was assessed using 22 questions that consisted of 6 questions on the pork tapeworm/taeniasis, 4 questions on HCC, 6 questions on PCC and 6 questions on human epilepsy. Similarly, attitude towards TSCT was investigated using 4 questions. The practice section contained 12 questions.

All practice-related questions had a value of 1 or 0 (correct response had a value of ‘1’ and a wrong or I don’t know response had a value of ‘0’). A cumulative practice score ranging from zero to 14 points was calculated for every participant and compared by district, demographic and socioeconomic factors using non-parametric tests (Mann–Whitney *U* test and Kruskal–Wallis test).

Before data collection, face validity was assessed in a pilot study on 10 respondents (7 pig- and 3 non-pig farmers) from a village outside the study area, to assess whether the questionnaire was understandable, clear, and easy to follow. The content validity was discussed among three experts (a research supervisor, a co-supervisor, and a veterinary expert) to decide whether or not the content of the questionnaire met the study objective.

### Direct household infrastructure observation

Direct observation and infrastructure assessment to record behaviour/hygiene practices associated with TSCT transmission were conducted using a checklist for each household. Direct observations focused on the presence and quality of toilets and pigpens, the presence of handwashing facilities inside or outside the latrines, pig management systems (confinement, tethering, or free-range), and general sanitation of the surroundings.

### Data collection

Data were collected using KoboCollect, the application for data collection through KoBoToolBox [28]. All three investigators were trained on the use of the KoboCollect tool before the commencement of the study; the investigators also pre-tested the feasibility and the correctness of the items in the electronic questionnaire. Data were collected by a team of three investigators (CN, CM, DM), the Ward Livestock Field Officer and the District Livestock and Fisheries Development Officer).

### Data analysis

Data were exported into a Microsoft Excel spreadsheet for cleaning and storage, and SPSS version 20.0 (Armonk, NY: IBM Corp), for statistical analysis. Descriptive statistics were applied through frequencies and percentages of correct responses. The Chi-square test was used to test for associations between categorical variables. Practice scores were compared by district, demographic and socioeconomic factors using non-parametric tests (Mann–Whitney U test and Kruskal–Wallis test). Data from household observation were also assessed by the Chi-square test. The significance level was p < 0.05.

## Results

483 participants were included in the study (121 in Mbulu District, 122 in Mpwapwa District, 121 in Mbinga District, and 119 in Rungwe District). 252 participants (52%) were males (Table 1). Most (340, 71%) respondents were older than 35 years. The main source of income for most respondents was livestock keeping (424, 88%); three quarters (367, 76%) of participants were pig keepers. The majority (384, 80%) of the respondents completed primary school education, while 47 (10%) had no formal education. Mbulu District had most of the large size households with above four members per household (57, 47%).

**Table 1:** Social demographic characteristics of respondents in four districts

		Mbulu	Mpwapwa	Rungwe	Mbinga	Total
		n (%)	n (%)	n (%)	n (%)	n (%)
		121	122	119	121	483
Sex	Male	56 (46)	63 (52)	74 (61)	59 (50)	252 (52)
	Female	65 (54)	59 (48)	47 (39)	60 (50)	231 (48)
Age Groups	18–25	24 (20)	11 (9)	8 (7)	10 (8)	53 (11)
	26–35	17 (14)	36 (30)	17 (14)	20 (17)	90 (19)
	36–50	46 (38)	49 (40)	43 (36)	44 (37)	182 (38)
	> 50	34 (28)	26 (21)	53 (44)	45 (38)	158 (33)
Period of Residence	Below 1	2 (2)	0 (0)	1 (1)	0 (0)	3 (1)
	1–5	12 (10)	5 (4)	5 (4)	13 (11)	35 (7)
	6–10	6 (5)	4 (3)	2 (2)	9 (8)	21 (4)
	Above 10	40 (33)	31 (25)	37 (31)	37 (31)	145 (30)
	All my life	61 (50)	82 (67)	76 (63)	60 (50)	279 (58)
Respondent Education	None	25 (21)	13 (11)	3 (3)	6 (5)	47 (10)
	Primary	86 (71)	101 (83)	102 (84)	95 (80)	384 (80)
	Secondary	6 (5)	7 (6)	14 (12)	14 (12)	41 (9)
	College	4 (3)	1 (1)	2 (2)	4 (3)	11 (2)
Main Occupation	Livestock keeping	117 (97)	100 (82)	108 (89)	99 (83)	424 (88)
	*Pig farmers*	100/117 (85)	92/100 (92)	75/108 (69)	88/99 (89)	367/424 (87)
	Fishing	0 (0)	0 (0)	0 (0)	0 (0)	0 (0)
	Business	4 (3)	24 (20)	9 (7)	19 (16)	56 (12)
	Other	4 (3)	9 (7)	5 (4)	7 (6)	25 (5)
Household Size	One	3 (3)	0 (0)	2 (2)	4 (3)	9 (2)
	2–3	10 (8)	8 (7)	12 (10)	16 (13)	46 (10)
	4–5	25 (21)	38 (31)	43 (36)	45 (38)	151 (31)
	6–7	26 (22)	46 (38)	38 (31)	41 (35)	151 (31)
	> 7	57 (47)	30 (25)	26 (22)	13 (11)	126 (26)
Major Problems in the village	Roads	23 (19)	23 (19)	68 (56)	26 (22)	140 (29)
	Water	37 (31)	73 (60)	20 (17)	65 (55)	195 (40)
	Electricity	55 (46)	31 (25)	53 (44)	40 (34)	179 (37)
	Dispensaries/Health centres	6 (5)	10 (8)	8 (7)	18 (15)	42 (9)
	Education	1 (1)	3 (3)	0 (0)	1 (1)	5 (1)
	Animal diseases	1 (1)	0 (0)	0 (0)	0 (0)	1 (0)
	Leadership	0 (0)	0 (0)	1 (1)	0 (0)	1 (0)
	No problems	7 (6)	1 (1)	0 (0)	1 (1)	9 (2)

Descriptive statistics for the KAP-related questions are presented in Tables 2 and 3. 233 (48%) respondents had heard about the pork tapeworm (*T. solium* taeniasis) and many of them (163, 65%) knew the signs/symptoms of an infection, but correct knowledge of transmission was low with only 13 (3%) respondents reporting transmission through poorly cooked pork; many (54, 23%) participants falsely cited contaminated water as the route of pork tapeworm infection. 129 (28%) were aware of the health effects of the pork tapeworm on humans, and 104 (22%) knew about control measures against pork tapeworm infection (Table 2, Additional file 1: Table S1A).

**Table 2:** Knowledge of respondents regarding *Taenia solium* taeniasis, porcine cysticercosis, and human cysticercosis

	Mbulu	Mpwapwa	Rungwe	Mbinga	Total	
	n (%)	n (%)	n (%)	n (%)	n (%)	p-value
	121	122	119	121	483	
Correct knowledge regarding pork tapeworm (*T. solium taeniasis*)						
Has heard about the pork tapeworm	54 (45)	57 (47)	63 (53)	59 (59)	233 (48)	0.61
Transmission of the pork tapeworm	4 (3)	2 (2)	2 (2)	5 (4)	13 (3)	0.55
Health effects on humans	18 (15)	26 (21)	40 (34)	45 (37)	129 (28)	< 0.001
Pork tapeworm treatment	50 (41)	46 (38)	53 (45)	49 (40)	198 (41)	0.25
Prevention of pork tapeworm infection	22 (18)	21 (17)	33 (28)	28 (23)	104 (22)	0.08
Correct knowledge regarding porcine cysticercosis (PCC)						
Has heard about PCC	119 (98)	119 (98)	107 (90)	121 (100)	466 (97)	< 0.001
Transmission of PCC	70 (58)	50 (41)	15 (13)	32 (26)	167 (35)	< 0.001
Prevention of PCC	43 (36)	90 (74)	69 (58)	108 (89)	218 (45)	< 0.001
Correct knowledge regarding human cysticercosis (HCC) and epilepsy						
Has heard about HCC	5 (4)	7 (6)	3 (3)	14 (12)	29 (6)	0.19
Transmission of HCC	7 (6)	5 (4)	2 (2)	6 (5)	20 (4)	0.42
Prevention of HCC	2 (2)	7 (6)	0 (0)	3 (2)	12 (2)	0.71
Has heard about human epilepsy	105 (87)	118 (97)	116 (97)	120 (99)	459 (95)	< 0.001

All p-values are based on a Chi-square analysis of numbers across the four districts

**Table 3:** Attitude and practices of respondents towards *Taenia solium* cysticercosis/taeniasis

	Mbulu	Mpwapwa	Rungwe	Mbinga	Total	
	n (%)	n (%)	n (%)	n (%)	n (%)	p-value
	121	122	119	121	483	
Attitude related questions (risk perceptions and measures against infected pork)						
Do you consider yourself at risk of being infected with tapeworm?—Yes	11/121 (9)	29/122 (24)	13/119 (11)	25/121 (21)	78/483 (16)	< 0.001
Are you at risk of being infected with cysticerci?—Yes	6/121 (5)	4/122 (3)	1/119 (1)	9/ (7)	20/483 (4)	< 0.001
Is it safe to eat pork infected with cysticerci?—No	44/121 (36)	30/122 (25)	53/119 (45)	14/121 (12)	141/483 (29)	< 0.001
Appropriate measures taken for infected pork—Yes	109/121 (90)	112/122 (92)	117/119 (98)	121/121 (100)	459/483 (95)	< 0.001
Practices related questions (acceptable preventive practices)						
Eats pork	109/121 (90)	93/122 (76)	84/119 (71)	106/121 (88)	392/483 (81)	< 0.001
Pig keeper	100/121 (83)	92/122 (75)	87/119 (73)	88/121 (73)	367/483 (76)	0.24
Pig confinement during farming season	65/100 (65)	92/92 (100)	75/83 (90)	88/88 (100)	320/367 (88)	< 0.001
Pig confinement after harvest	82/100 (82)	88/92 (96)	83/83 (100)	88/88 (100)	341/367 (94)	< 0.001
Free-range pigs are seen as potential cause of infection	86/121 (71)	90/100 (90)	69/87 (79)	81/88 (92)	326/396 (82)	< 0.001
Ever slaughtered a pig in the backyard	26/121 (21)	19/122 (16)	8/119 (7)	41/121 (34)	94/483 (19)	< 0.001
If yes, meat was inspected	7/26 (27)	19/19 (100)	6/8 (75)	35/41 (85)	67/94 (71)	< 0.001
Participant can identify cysticerci	99/109 (91)	73/93 (79)	59/84 (70)	98/106 (92)	329/392 (84)	< 0.001
Always cooks pork properly	72/104 (69)	51/81 (63)	69/81 (85)	81/99 (82)	273/365 (75)	0.002
Always washes vegetables and fruits before eating raw	112/121 (93)	119/122 (98)	114/119 (96)	120/121 (99)	465/483 (96)	0.04
Always washes hands with soap before eating	68/120 (57)	87/120 (72)	47/116 (40)	49/121 (40)	251/477 (53)	< 0.001
Always treats drinking water	37/121 (31)	44/122 (36)	57/119 (48)	56 /121(46)	194/483 (40)	0.02
Always uses toilet/latrine	117/121 (97)	122/122 (100)	118/119 (99)	121/121 (100)	478/483 (99)	0.03
Always washes hands with soap after toilet use	74/94 (79)	92/103 (89)	73/101 (72)	74/99 (75)	313/397 (79)	0.02

All p-values are based on a Chi-square analysis of numbers across the four districts

Most (466, 97%) respondents had heard about PCC, every third (n = 167) knew the correct transmission of PCC and every other participant (218, 45%) knew control measures against PCC. The last two questions were more commonly known by pig farmers (data not presented). For all these variables, knowledge varied by district (p < 0.001 for all three; Table 2, Additional file 1: Table S1B). Most participants had never heard of HCC, hence knowledge about the transmission or HCC preventive measures was poor too. Many people had heard about and knew the signs/symptoms of human epilepsy. About 364 (75%) of the participants knew a person with epilepsy, and 64 (13%) had a family member with epilepsy. However, the link between the pork tapeworm and epileptics seizures was mostly unknown (Table 2, Additional file 1: Table S1d). The source of information for all aspects of TSCT was mainly friends and family members. Health information at school, health centre or through radio and TV were only seldomly mentioned (Additional file 1: Table S1A–D).

Risk perception of infection with the pork tapeworm and the development of cysticercosis was generally very low (20; 4%) and varied between districts (p < 0.001 for both questions). In Rungwe, the district with unknown *T. solium* endemicity, the risk perception was particularly low. Overall, 71% of the participants said it was safe and 29% said it was not safe to eat infected pork, but nearly all participants reported taking appropriate measures whenever they saw the infected pork, and these include reporting to the Veterinarian/Livestock Extension Officers and neither selling nor eating the pork (Table 3). Three hundred ninety-two (81%) of the participants ranging from 90% in Mbulu to only 71% in Rungwe (p < 0.001) ate pork. As for the practices, 367 (76%) of the respondents were pig keepers and the proportion was evenly distributed across the districts (p = 0.24). Most pig keepers, 320 (88%) and 341 (94%) reported confining their pigs during farming season and after harvest respectively, to avoid a penalty/fine or to protect crops and the environment (Table 3). Every fifth pig keeper had slaughtered pigs in their backyard and mostly the pork was inspected after the slaughtering except for Mbulu where only 27% reported meat inspection. Overall, 319, that is, 83% of the participants reported knowing what cysticerci looked like, but this proportion differed by district (p < 0.001). Rungwe had the lowest (59, 70%) whereas Mbinga had the highest (98, 91%) proportion. Whilst most participants reported washing their vegetables/fruit before eating, many reported not cooking pork properly. Also, 251 (53%) participants reported washing their hands before eating, and 313 (79%) after using the toilet (Table 3). Overall, the practice score was higher for males than was for females and for pig farmers than was for non-pig farmers; the score increased with education level and differed by district, but was not dependent on age (Additional file 1: Table S2).

### Household infrastructures observation across the four districts

**Fig. 2:**
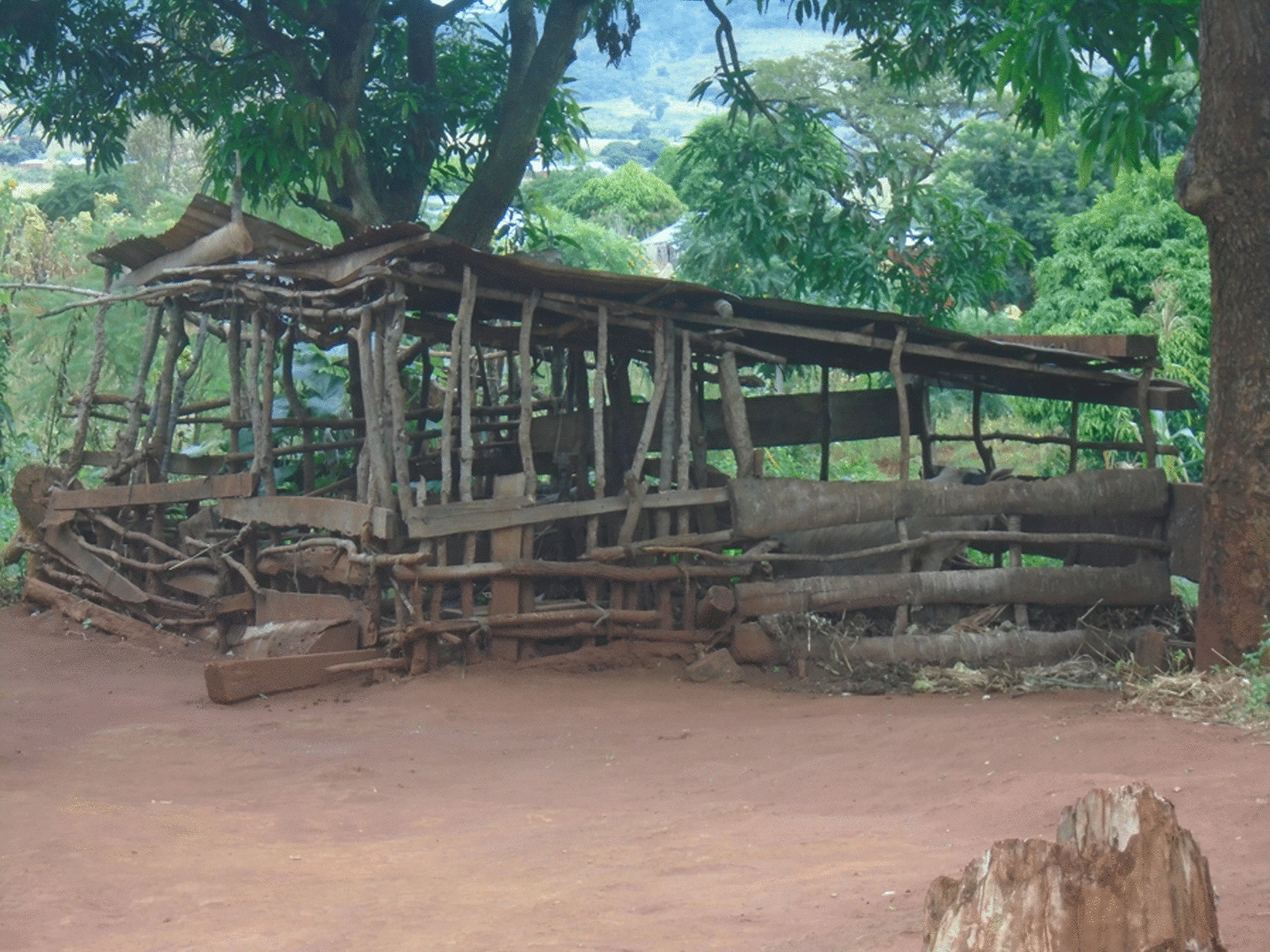
Pigpen construction with clay floor and wood fence

Household observations revealed that 362 or 78% of the households had toilets, with four out of five being latrines (274, 80%) and only 20% being flush/pour toilets (69; Table 4). Sanitation in the households was poor with a lack of functional handwashing facilities and poor latrines without cemented floors. Floors in most of the toilets were constructed using sand/clay (219, 64%), and most toilets (272, 78%) had a complete roof. More than half of all toilets had no handwashing facilities. Concerning pig management, 234, that is, 69percent of the households had a pigpen which most commonly had a sand/clay floor except for Mbinda District where most of the pigpens had cement floor (Table 4, Fig. 2).

**Table 4:** Household infrastructures observation

		Mbulu	Mpwapwa	Rungwe	Mbinga	Total	
		n (%)	n (%)	n (%)	n (%)	n (%)	p-value
		121	122	119	121	483	
Households with toilet		90/120 (67)	100/110 (91)	101/117 (86)	71/115 (62)	362/462 (78)	< 0.001
Complete toilet wall		75/90 (83)	98/100 (98)	100/101 (99)	68/71 (96)	341/362 (94)	< 0.001
Complete toilet roof		65/86 (76)	75/94 (80)	76/95 (80)	56/69 (81)	272/344 (79)	0.850
Toilet floor material	Clay/sand	70/86 (81)	67/94 (71)	52/95 (55)	30/69 (43)	219/344 (64)	< 0.001
	Cement	15/86 (17)	27/94 (29)	35/95 (37)	34/69 (49)	111/344 (32)	
	Wood/tiles	1/86 (1)	0/94 (0)	8/95 (8)	5/69 (7)	14/344 (4)	
Type of toilet	Flushing/pour toilet	3/84 (4)	10/97 (10)	31/95 (33)	25/68 (37)	69/343 (20)	< 0.001
	Pit latrine	81/84 (96)	87/97 (90)	64/95 (67)	43/68 (63)	274/343 (80)	
Faeces seen on toilet floor		1/86 (1)	1/94 (1)	1/95 (1)	0/69 (0)	3/344 (1)	0.577
Hand washing facilities in toilet	Yes, ≤ 5 m	19/86 (22)	20/94 (21)	44/95 (46)	33/69 (48)	116/344 (34)	< 0.001
	Yes, > 5 m	2/86 (2)	2/94 (2)	0/95 (0)	0/69 (0)	4/344 (1)	
	No hand washing facilities	65/86 (76)	60/94 (64)	52/95 (55)	36/69 (52)	213/344 (62)	
Hand washing soap in toilet		11/86 (13)	14/94 (15)	11/95 (12)	1/69 (1)	37/344 (11)	< 0.001
Presence of pigpen in the household		65/120 (54)	83/110 (75)	77/117 (66)	68/115 (59)	234/462 (51)	0.012
Pigpen floor materials	Sand/clay	56/65 (86)	73/87 (88)	59/77 (77)	9/68 (13)	197/234 (67)	< 0.001
	Cement	2/65 (3)	6/83 (7)	2/77 (3)	33/68 (49)	43/234 (15)	
	Wood	4/65 (6)	4/83 (5)	13/77 (17)	26/68 (38)	47/234 (16)	
	Other materials	3/65 (4)	0/83 (0)	3/77 (3)	0/68 (0)	6/234 (2)	

All p-values are based on a Chi-square analysis of numbers across the four districts

## Discussion

In this study, we evaluated knowledge, attitudes and practices towards TSCT in rural Tanzania, by a combined assessment through a questionnaire survey and households inspection. Interestingly, almost all the participants had heard about PCC, half of the participants knew about the pork tapeworm (*T. solium* taeniasis), but almost nobody had heard anything about HCC. The questionnaire also revealed that knowledge of the means of transmission and the various aspects of pork tapeworm and HCC prevention was particularly poor, although the latter is not surprising as no one had ever heard anything about HCC. The knowledge on the transmission and prevention of PCC was fair in the aforementioned areas. The effects of *T. solium* on human health (taeniasis and NCC) were not well known, although knowledge of signs/symptoms of taeniasis was fair, compared to knowledge of the effects on pig health which were mainly known among pig keepers. The general good knowledge about PCC may be attributable to epidemiological studies on PCC conducted previously in the districts (25,26) and information obtained through village meetings/gatherings, household visits by Extension Officers or leaflets. Not only was the knowledge on the impact of *T. solium* on human health and HCC in general limited, but also many participants did not consider themselves at risk of *T. solium* infection, despite living in areas with high *T. solium* endemicity and having heard about the parasite before, at least in the context of *T. solium* taeniasis. This was surprising because several studies on *T. solium,* with different research questions, had previously been conducted in some of the study districts [6, 18, 20, 22–24, 29, 30]; among these studies were also studies that assessed appropriate *T. solium* health education packages which proved successful. This means that health education packages may not have had sustained effects on knowledge and practices regarding TSCT and that new health education packages not only need to deliver information on *T. solium* but also need to include the risk perception of participants. Health education packages, therefore, have to be perceived as an iterative process including not only the researchers and the local authorities but also and foremost the local communities from the beginning and throughout the entire process in a co-creative design.

To the best of our knowledge, to date, no studies regarding TSCT have been conducted in the Rungwe district. Nonetheless, participants from Rungwe showed similar results to those shown by participants in other districts in all KAP domains. This may be a result of good extension services and other interventions on sanitation and hygiene in this district. This could also be attributable to knowledge spill over from the adjacent districts that are known to be PCC endemic thus representing a desirable effect. In addition, the district is popular for intensive dairy cattle management which may further explain their pig management system and pig feeding practices. Thus, knowledge transfer from one knowledge domain to another should not be underestimated, instead, it should be explored in health education programmes.

On average, pig keepers knew that free-roaming of pigs and exposure to human faeces and contaminated feeds were central to the transmission of *T. solium* infection. This finding is comparable to the findings from several other studies conducted in Tanzania [8, 9, 24, 25]. On the contrary, most respondents had never heard of HCC, possibly due to a lack of knowledge among medical staff and/or lack of appropriate diagnostic tools. It seems that people are hardly ever diagnosed with HCC (especially NCC) outside research projects in sub-Saharan Africa as access to neuroimaging facilities is mandatory for the correct diagnosis of NCC but very costly. However, our findings are not unusual. Also, other studies conducted in areas highly endemic to *T. solium* showed low awareness of HCC/NCC [18, 31, 32]. Interestingly, knowledge about epilepsy was high, but not many people knew that *T. solium* cysticercosis is one of the causes of epilepsy.

In general, the level of knowledge on PCC was good. This may be because almost three-quarters of the respondents were pig farmers who might have acquired their knowledge from Livestock Field Officers (LFO) and other extension agents allocated to their respective villages. In the present study, most participants, especially pig farmers were aware of the predilection sites of *T. solium* cysticerci in the infected pig; under the tongue being the most commonly mentioned site. This may be because when selling pigs to traders, they usually check for the presence of cysticerci under the tongue. Regarding the aetiology of PCC, some interesting misconceptions persist. Pawpaw seeds and jiggers (sand fleas) were reported by a few farmers as causes of PCC, all of the farmers were from Rungwe District where feeding fruits to pigs is a very common practice (results not presented). This is probably because of the similarity in the morphology of *T. solium* cysticerci to pawpaw seeds and jiggers (sand fleas). This confusion implies that although general knowledge of PCC was good, there is still a need for health education to consolidate knowledge even among pig farmers.

In our study, most of the pig farmers visited had access to veterinary services and were frequently visited by LFO. This is likely the reason, why pig farmers had often higher knowledge about *T. sodium* related health questions. This may indicate access to some education interventions that had already been conducted before. Information for farmers is mostly provided by LFO through village meetings, and sometimes through household visits during the treatment of pigs, usually focusing on PCC and other animal-related diseases. LFOs usually encourage farmers to confine their pigs without including proper information on feeding and/or TSCT control/elimination, in general. Likewise, it is customary for medical practitioners in health facilities to provide education on environmental sanitation, personal hygiene, and human health-related matters. In most cases, only patients and expectant mothers visiting health facilities for other medical services benefit from such education, while the rest of the community members remain uninformed. This rather siloed approach calls for a One Health perspective, that is, the close collaboration of medical doctors and nurses, veterinarians, and LFO together with environmental and sanitation officers in addressing the matter. There is a need for joint training and information campaigns in the communities to provide cross-sectoral information on TSCT. Ultimately, the One Health collaboration should not only concern communities at the grassroots level but also relevant policymakers at the district, regional and national levels.

Practices reported by the respondents during our questionnaire survey often differed from those observed during direct household observations. While the majority of the respondents reported always using the toilet, household observations found many households without toilets. One of the striking observations was that in some of the visited households, children’s faeces was disposed of directly into pigpens, which enhances the completion of the *T. solium* life cycle. Three-quarters of the respondents reported washing their hands with water and soap after using the toilet, but household observations found that less than half of all toilets had installed handwashing facilities, commonly known as tippy taps; but most of them were without water or soap on grounds that the tippy taps were emptied to safeguard the health of children who used to play with them and drink the water from the handwashing facilities. Furthermore, while the majority of the respondents reported confining their pigs, nearly half of the households had either no pigpens or pigpens with spaces for pigs, especially piglets, to escape and roam freely. The discrepancy between questionnaire responses and household observations shows the importance of direct observation in assessing KAP as many questions may be given socially desired responses.

Our findings are not only relevant for local stakeholders but also for the overall target of control/elimination of TSCT as outlined in the WHO roadmap for neglected tropical diseases 2021–2030 [33]. The roadmap also mentions four cross-cutting targets for 2030 which include (1) integrated approaches, (2) multisectoral coordination, (3) universal health coverage and (4) country ownership. Other important points made include the involvement of local communities by social mobilisation at the very basis of every health intervention, knowledge sharing, and prevention strategies targeted at local situations. The current project is following those principles, and in a second step, a contextualised and co-created health education intervention will be developed. The WHO roadmap for neglected tropical diseases also emphasizes the integrated approaches and multisectoral coordination which calls for a One Health approach to the plan. Our study demonstrated that knowledge was rather different between PCC and HCC/NCC which shows clear potential for the engagement of the humans, animals and environmental sectors beyond their boundaries, that is, medical officers informing about the relevant animal diseases and LFO informing about human diseases. In addition, environmental services need to be promoted and brought into the big picture as most transmission happens because of a lack of hygiene and within various environmental compartments. However, this also requires adopting a “whole of the system” approach, where the human, animal and environmental sectors need to be strengthened in an equitable manner, which, in turn, requires good One Health governance at different levels. The objectives and results of our study contribute to the educational One Health approach of TSCT in the control/elimination and prevention. Although community-based TSCT health education has shown to have an impact on HCC and PCC [34, 35], the integration of different TSCT One Health approaches, including treatment of PCC and taeniasis, the prevention of PCC by vaccination and health education, seem to be able to fully eliminate active PCC [35].

## Strengths and limitations

The strength of this study is its large sample size of almost 500 households, that comprehensive knowledge about TSCT was evaluated and that the answers were checked by household infrastructure observation. In surveys, the respondents tend to respond to questions in a socially desirable way. Conducting household infrastructure observations, we were able to confirm or discard some of the responses to the questionnaire. This was not only important for the quality of the results of the current paper but also for the health education package which was developed based on the results of this study.

Our study also had limitations. One limitation was that although a pilot study was conducted only 10 households were included due to logistic reasons and time constraints. Another limitation was that our questionnaire used pre-specified answers which allowed the guessing of correct answers. However, we controlled this by using enumerators during the household survey. The enumerators asked questions without first revealing the possible answers. Another limitation was that the questionnaire was administered by three different people, but as answers were prespecified, this may not have had a large effect. Also, only one person per household was interviewed which may have pre-selected people who are more knowledgeable about TSCT. The degree of this bias, however, could not be verified. In addition, we were able to identify gaps in knowledge about the link between TSCT and human disease, but we neither assessed the prevalence of PCC nor HCC in the study districts. For PCC, three of the four districts were known to be highly endemic for *T. solium*, but not necessarily the villages we studied. PCC and HCC were likely prevalent in the study villages, but as we did not investigate this we were not able to establish the relationship between low or incorrect KAP and high prevalence of PCC and/or HCC. However, the evaluation of the impact of KAP and an especially designed community-based health education package (for explanation see above) on the presence or absence of PCC/HCC within the large health consortium of CYSTINET-Africa is currently underway and the results will most likely be presented towards the end of the year.

## Conclusions and recommendation

This study has revealed poor knowledge of TSCT with generally better knowledge about aspects related to pig health compared to the aspects related to human health. There was fair knowledge of epilepsy but the connection between NCC and epilepsy was not made. The limited general knowledge and negative practices (although the overall practice score was acceptable) may represent an important barrier to the control and elimination efforts of TSCT. It is, therefore, necessary to scale up the efforts in knowledge sharing with the public on transmission, TSCT signs/symptoms, control, treatment and prevention in Tanzania, preferably in a One Health approach, for the control and eradication of TSCT. The results from our study influence the design of context-specific health education packages that may help reach some of the goals specified in the WHO roadmap for neglected tropical diseases 2021–2030.

## Supplementary Information

**Additional file 1: Table S1A.** Knowledge about pork tapeworm (*T. solium taeniasis*)**. Table S1B.** Knowledge about porcine cysticercosis**. Table S1C.** Knowledge about human cysticercosis**. Table S1D.** Knowledge about epilepsy**. Table S2.** Practice score of Taenia solium taeniasis/cysticercosis (TSCT) by specific variables**.** Questionnaire

## Data Availability

The data used and/or analysed during the current study as well as any extra materials are not publicly available due to being part of an ongoing study but are available from the corresponding author upon reasonable request.

## Abbreviations

TSCT: *Taenia **solium* cysticercosis/taeniasis
KAP: Knowledge, attitude and practices
LFO: Livestock field officers
Ag-ELISA: Antigen-based enzyme-linked immunosorbent assay
HCC: Human cysticercosis
NCC: Neurocysticercosis
PCC: Porcine cysticercosis
WHO: World Health Organization
