# Supplementary material for: Knowledge, attitudes and practices related to Taenia solium cysticercosis and taeniasis in Tanzania
Source: BMC Infect Dis. 2022 Jun 13;22:534. doi: 10.1186/s12879-022-07408-0 (PMC9190087; doi:10.1186/s12879-022-07408-0)
Supplement: Supplementary file 1 — Additional file 1: Table S1A. Knowledge about pork tapeworm (T. solium taeniasis). Table S1B. Knowledge about porcine cysticercosis. Table S1C. Knowledge about human cysticercosis. Table S1D. Knowledge about epilepsy. Table S2. Practice score of Taenia solium taeniasis/cysticercosis (TSCT) by specific variables. Questionnaire [file 12879_2022_7408_MOESM1_ESM.docx]

**- SUPPLEMENTARY MATERIAL -**

**Knowledge, Attitudes and Practices related to *Taenia solium* Cysticercosis and Taeniasis in Tanzania**

**Chacha Nyangi^1,2^*, Dominik Stelzle^3,4^, Ernatus M Mkupasi^1^, Helena A Ngowi H^1^, Ayubu J Churi^5^, Veronika Schmidt^3,6^, Christopher Mahonge^7^, Andrea S Winkler^3,6^**

**Correspondence email: andrea.winkler@tum.de*

*1 Department of Veterinary Medicine and Public Health, Sokoine University of Agriculture,*

*Morogoro, Tanzania*

*2 Department of Applied Sciences, Mbeya University of Science and Technology, Mbeya, Tanzania*

*3 Center for Global Health, Department of Neurology, Technical University of Munich (TUM), Germany*

*4 Chair of Epidemiology, Department of Sports and Health Sciences, Technical University Munich (TUM), Germany*

*5 Centre for Information and Communication on Technology (CICT), Sokoine University of Agriculture, Morogoro, Tanzania*

*6 Centre for Global Health, Institute of Health and Society, University of Oslo, Norway*

*7 Department of Policy, Planning and Management, Sokoine University of Agriculture, Morogoro, Tanzania*

**Supplementary Table 1A. Knowledge about pork tapeworm (*T. solium* taeniasis)**

|  |  | **Pork tapeworm (*T. solium* taeniasis)** | |
| --- | --- | --- | --- |
|  |  | **n** | **%** |
| Has heard of pork tapeworm *T. solium* | | 233/483 | 48% |
| Source of information | Friends | 55 | 24% |
|  | Brochures | 2 | 1% |
|  | Radio | 0 | 0% |
|  | Health centre | 78 | 34% |
|  | School | 1 | 0% |
|  | Parents | 0 | 0% |
|  | No single specific source | 81 | 35% |
|  | Other source | 32 | 14% |
| Way of tapeworm transmission | Poorly prepared pig meat | 13 | 3% |
|  | Taking contaminated water/food | 54 | 11% |
|  | Other sources | 42 | 9% |
|  | Not aware | 124 | 26% |
| Awareness of health effects of tapeworm in human | Yes | 129 | 28% |
|  | No/ Not aware | 354 | 72% |
| Tapeworm signs/symptoms | Incorrect/ Not aware | 88 | 35% |
|  | Correct | 163 | 65% |
|  | *Loosing weight* | *47* | *19%* |
|  | *Diarrhoea* | *64* | *25%* |
|  | *Stomach swelling* | *60* | *24%* |
|  | *Poor growth* | *14* | *6%* |
|  | *Loosing apetite* | *85* | *34%* |
|  | *Stomach ache* | *76* | *30%* |
|  | *Other symptoms* | *84* | *33%* |
| Possibility of getting tapeworm in the surroundings | Yes | 78 | 16% |
|  | No/ Not aware | 405 | 84% |
| Can the tapeworm be treated? | Yes | 198 | 79% |
|  | No/ Not aware | 54 | 21% |
| Which is the correct treatment? | Don't know | 108 | 55% |
|  | *Anthelmintics* | *83* | *38%* |
|  | *Other methods* | *28* | *14%* |

**Supplementary Table 1B. Knowledge about porcine cysticercosis**

|  |  | **PCC** | |
| --- | --- | --- | --- |
|  |  | **n** | **%** |
| Has heard of PCC |  | 466/483 | 96% |
| Source of information | Friends | 103 | 22% |
|  | Brochures | 1 | 0% |
|  | Radio | 0 | 0% |
|  | Health centre | 33 | 7% |
|  | School | 1 | 0% |
|  | Parents | 5 | 1% |
|  | No single specific source | 307 | 66% |
|  | Other source | 24 | 5% |
| Way of PCC transmission | Correct | 167 | 36% |
|  | Incorrect/Does not know | 316 | 64% |
| Location of cysts^⸸^ | Meat | 204 | 47% |
|  | Under the tongue | 236 | 54% |
|  | Brain | 12 | 3% |
|  | Heart | 37 | 8% |
|  | Intestine | 34 | 8% |
|  | Neck | 169 | 39% |
|  | Other part of the body | 92 | 21% |
| Awareness of health effects of PCC in pigs | Yes | 199 | 43% |
|  | No/ Not Aware | 267 | 57% |
| Suitable PCC control method | Contact veterinary doctor | 218 | 47% |
|  | Use local method | 16 | 3% |
|  | Slaughtering and burn | 91 | 20% |
|  | Other method | 118 | 25% |
|  | Don't know | 23 | 5% |

^⸸^ More than one answer possible

**Supplementary Table 1C. Knowledge about human cysticercosis**

|  |  | **HCC** | |
| --- | --- | --- | --- |
|  |  | **n** | **%** |
| Has heard of human cysticercosis | | 29/483 | 6% |
| Source of information | Friends | 19 | 66% |
|  | Brochures | 1 | 3% |
|  | Radio | 2 | 7% |
|  | Health centre | 2 | 7% |
|  | School | 0 | 0% |
|  | Parents | 0 | 0% |
|  | No single specific source | 0 | 0% |
|  | Other source | 6 | 21% |
| Way of HCC transmission | Poorly preaperd veg/fruits | 3 | 2% |
|  | Contaminated water/food | 5 | 4% |
|  | Use of contaminated hands | 2 | 2% |
|  | Poorly prepared pig meat | 11 | 9% |
|  | Not aware | 102 | 83% |
| Awareness health effects of HCC in humans | Yes | 34 | 28% |
|  | No/ Not aware | 89 | 72% |
| Suitable HCC control | Yes | 37 | 60% |
|  | No/ Not aware | 25 | 40% |

**Supplementary Table 1D. Knowledge about epilepsy**

|  |  | **Epilepsy** | |
| --- | --- | --- | --- |
|  |  | **n** | **%** |
| Has heard of epilepsy |  | 459/483 | 95% |
| Source of information | Friends | 86 | 19% |
|  | TV | 5 | 1% |
|  | Radio | 7 | 2% |
|  | Health centre | 24 | 5% |
|  | School | 10 | 2% |
|  | Parents | 301 | 66% |
|  | No specific source | 0 | 0% |
|  | Other source | 62 | 14% |
| Knows a person with epilepsy | | 364 | 75% |
| Family member with epilepsy | | 64 | 13% |
| Signs/symptoms of epilepsy | Tiredness | 56 | 12% |
|  | Unconsciousness and uncontrolable shaking | 371 | 77% |
|  | Froth from the mouth | 295 | 61% |
|  | Other symptoms | 153 | 32% |
|  | Don't know | 73 | 15% |
| Cause of epilepsy | Correct | 29 | 6.4% |
|  | Incorrect/Don't know | 424 | 93.6% |
| Cause of epilepsy detailed | Contaminated food/water | 3 | 0.7% |
|  | Contaminated vegetables/fruits | 1 | 0.2% |
|  | Inherited from parents | 25 | 5.5% |
|  | Poorly prepared pork | 13 | 2.9% |
|  | Being bewitched | 25 | 5.5% |
|  | Other | 22 | 4.9% |
| Prevention of neurocysticercosis | Correct method | 12 | 2% |
|  | Incorrect method/Does not know | 471 | 98% |

**Supplementary Table 2: Practice score of *Taenia solium* taeniasis/cysticercosis (TSCT) by specific variables**

| **Variable** | **Practice total score**  **Mean (95%CI)** |  |
| --- | --- | --- |
| **Sex** | | |
| Male | 10.8 (9.3-11.8) | P<0.001* |
| Female | 9.8 (8.6-11) |  |
| **Education** | | |
| None | 8.6 (7.6-10.2) | P<0.001** |
| Primary | 10.5 (8.9-11.8) |  |
| Secondary | 10.8 (9.5-11.8) |  |
| Tertiary | 11.8 (10.5-13) |  |
| **Districts** | | |
| Mbulu | 9.7 (8.6-11.2) | P<0.001** |
| Mpwapwa | 10.8 (9.3-11.8) |  |
| Rungwe | 9.7 (8.6-11.0) |  |
| Mbinga | 10.8 (9.7-11.8) |  |
| **Age group** | | |
| 18-25 | 10 (8.8-10.8) | P=0.38** |
| 26-34 | 10.5 (10.5-11.8) |  |
| 35-45 | 10.5 (8.9-11.8) |  |
| 46+ | 10.5 (8.8-11.8) |  |
| **Occupation** | | |
| Farming | 8.8 (6.2-9.8) | P<0.001** |
| Livestock keeping | 10.5 (9.3-11.8) |  |
| Business and livestock keeping | 10.8 (9.3-11.8) |  |
| **Household size** | | |
| 1 to 2 | 9.8 (8.9-11.5) | P = 0.84** |
| 3 to 4 | 10.5 (8.8-11.8) |  |
| 5+ | 10.3 (8.8-11.8) |  |
| **Keeping pigs** | | |
| No pig farmer | 8.6 (7-9.8) | P<0.001* |
| Pig farmer | 10.8 (9.7-11.8) |  |

*Values are statistically significant at p<0.05;*

*Mann-Whitney U test;

**Kruskal-Wallis test;

**Supplementary Figure. Picture**


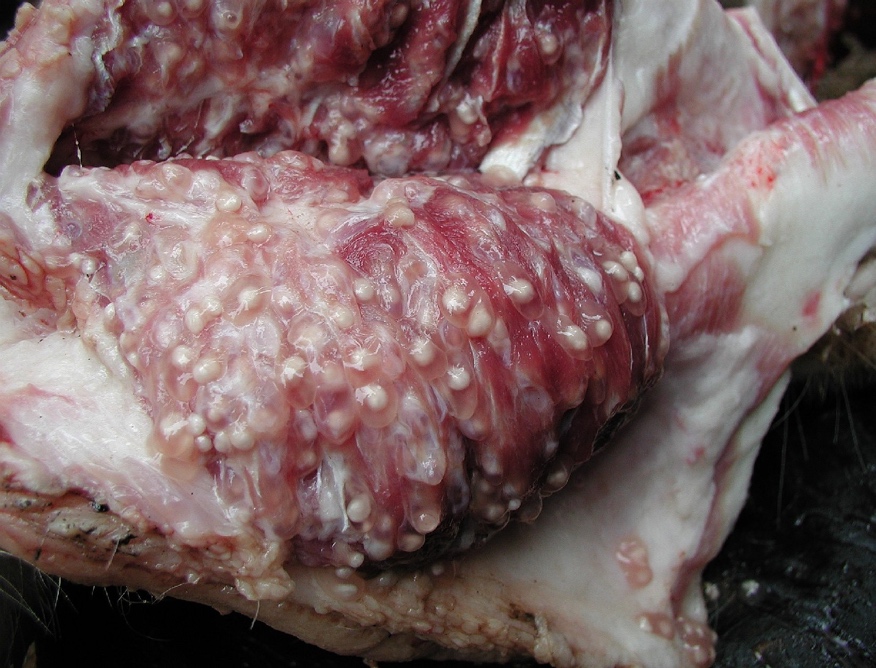


Source: Willingham AL. 2008. Combating *Taenia solium* Cysticercosis in Southeast Asia: An Opportunity for Improving Human Health and Livestock Production. Departement of Veterinary Pathology, University of Copenhagen

**Supplementary file. Questionnaire**

**Personal Information of the Respondent**

1. Sex

(1) Male

(2) Female

2. Age (years) ………………

**Background & Village**

1. How long have you been living in this village (years)?

(1) < 1 year

(2) 1 – 5 years

(3) 6 – 10 years

(4) > 10 years

(5) All my life

2. What’s the most serious health problem in your village? (choose one answer)

(1) Diarrhoea

(2) Malnutrition

(3) Heart diseases

(4) Abdominal pain

(5) Intestinal worms

(6) Malaria

(7) Cholera

(8) Others

If others, specify ……………………………………………………………

**Socio-Economic Status**

1. Level of Education

(1) No formal education

(2) Primary school education

(3) Secondary school education

(4) Tertiary education

2. Occupation (more than one answer possible)

(1) Smallholder farmer

(2) Livestock keeping

(3) Business

(4) Fishing

(5) Others

If others, specify ……………………………………………………………

3. How many people (including yourself) are there in your household?

(1) Alone

(2) 2 – 3

(3) 4-5

(4) 6-7

(5) >7

**Knowledge on Transmission, Symptoms, Treatment and Prevention**

1. Have you heard about the intestinal tapeworm called *T. solium*? (show picture of pork tapeworm)

(1) Yes

(2) No

If yes, where have you heard of it? (more than one answer possible)

(1) Friend

(2) Poster

(3) Radio

(4) Health centers

(5) School

(6) Parents/family

(7) Seen myself

(8) Other sources

If other sources, specify ……………………………………………………………

2. How do you think the intestinal tapeworm (*T. solium*) infection is acquired? (choose one answer)

(1) By eating raw/undercooked meat infected with cyst

(2) By drinking contaminated water

(3) Don’t know

(4) Others

If others, specify ……………………………………………………………

3. Do you think the intestinal worm (*T. solium*) can cause health problems to human?

(1) Yes

(2) No

(3) Don’t know

If others, specify ……………………………………………………………

4. What are the symptoms that occur in people infected with the intestinal worm (*T. solium*) (more than one answer is possible)

(1) Vision impairment

(2) Weight loss

(3) Diarrhoea

(4) Stomach swelling

(5) Growth retardation

(6) Poor appetite

(7) Abdominal discomfort

(8) Others

(9) Don’t know

If others, specify ……………………………………………………………

5. Do you think the intestinal worm (*T. solium*) can be treated?

(1) Yes

(2) No

(3) Don’t know

If yes, what are the medicine for the treatment of *T. solium* (more than 1 answer possible)

(1) Mebendazole

(2) Albendazole

(3) Praziquantel

(4) Niclosamide

(5) Don’t know

(6) Others

If others, specify ……………………………………………………………

6. Is there any way to prevent someone from getting the intestinal pork tapeworm (*T. solium*)?

(1) Yes

(2) No

(3) I don’t know

If yes, specify ……………………………………………………………

7. Have you heard about human cysticercosis (HCC)?

(1) Yes

(2) No

(3) Don’t know

If you have heard about human cysticercosis, where have you heard? (more than one answer possible)

(1) Friend

(2) Poster

(3) Radio

(4) Health centers

(5) School

(6) Parents/family

(7) Seen myself

(8) Other

If others, specify ……………………………………………………………

8. How can a human being acquire cysticercosis? (more than one answer possible)

(1) Unwashed fruits and vegetables

(2) Food/water contaminated with tapeworm eggs

(3) Not washing hands after defecating or before eating

(4) Eating undercooked/raw infected pork

(5) Don’t know

If others, specify ……………………………………………………………

9. Do you think human cysticercosis can cause serious health problems?

(1) Yes

(2) No

(3) Don’t know

If yes, specify ……………………………………………………………

10. Is there any way to prevent someone from getting cysticercosis?

(1) Yes

(2) No

(3) I don’t know

If yes, specify ……………………………………………………………

11. Have you heard about porcine cysticercosis (PC)?

(1) Yes

(2) No

If you have heard about porcine cysticercosis, where have you heard? (more than one answer possible)

(1) Friend

(2) Poster

(3) Radio

(4) Slaughterhouse/slab

(5) School

(6) Parents/family

(7) Seen myself

(8) Others

If others, specify ……………………………………………………………

12. How can a pig acquire cysticercosis?

(1) Feeding on contaminated feed/water

(2) Eating contaminated human faeces

(3) Others

(4) Don’t know

If others, specify ……………………………………………………………

13. What are the locations for cysts in pigs? (more than one answer possible)

(1) Muscle

(2) Under the tongue

(3) Brain

(4) Heart

(5) Stomach

(6) Neck muscles

(7) Others

If others, specify ……………………………………………………………

14. What measures do you take if you discover your pigs have cysticercosis? (more than one answer possible)

(1) Seek veterinary advice

(2) Use traditional medicine

(3) Slaughter and burry/burn

(4) Other measures

(5) Don’t know

If traditional medicine, specify ……………………………………

If other measures, specify …………………………………………

15. Do you think porcine cysticercosis can cause health effects to pigs?

(1) Yes

(2) No

(3) Don’t know

If yes, specify ……………………………………………………………

16. Is there any way to prevent pigs from getting cysticercosis?

(1) Yes

(2) No

(3) I don’t know

If yes, specify ……………………………………………………………

17. Have you heard about human epilepsy?

(1) Yes

(2) No

If yes, where have you heard? (more than one answer possible)

(1) Friend

(2) TV

(3) Radio

(4) Health centers

(5) School

(6) Parents/family

(7) Seen myself

(8) Others

If others, specify where ……………………………………………………………

18. How can a human being acquire epilepsy? (more than one answer possible)

(1) Food/ water contaminated with tapeworm eggs

(2) Unwashed fresh vegetables fruits

(3) Eating undercooked/raw infected pork

(4) Inheritance from parents

(5) Witchcraft

(6) Others

(7) Don’t know

If others, specify how ……………………………………………………………

19. Have you heard or seen a person with epilepsy in the village?

(1) Yes

(2) No

If yes, approximately how many? …………

20. What are the symptoms/signs of people suffering from epilepsy? (more than one answer possible)

(1) Feeling tired/dizziness

(2) Blindness

(3) Seizures

(4) Frothing in mouth

(5) Severe headache

(6) Others

(7) Don’t know

If others, specify ……………………………………………………………

21. Do you know the control measures against human epilepsy?

(1) Yes

(2) No

(3) Don’t know

If yes, which of the following are correct?

(1) Personal/environmental hygiene & sanitation

(2) Proper cooking of pork

(3) Proper use and maintenance of toilets

(4) Washing fruits and vegetables

(5) Drinking clean and safe water

(6) Treatment (deworming)

(7) Spiritual prayer

(8) Don’t know

(9) Others

If others, specify ……………………………………………………………

22. Have you or anyone of your family members suffered from epilepsy?

(1) Yes

(2) No

If yes, did you/they receive treatment?

(1) Yes

(2) No

If yes, where did you/they go for treatment?

(1) Hospital

(2) Used traditional medicine

(3) Other

(4) Not treated

Give reason(s) for choosing the type of treatment ………………………….

**Attitudes**

1. Do you think you are at the risk of being infected with the intestinal tapeworm (*T. solium*).

(1) Yes

(2) No

(3) Don’t know

If yes, mention the risk factors ......…………………………………………

If no, give reasons ……………......…………………………………………

2. Do you think you are at the risk of being infected with cysticerci?

(1) Yes

(2) No

(3) Don’t know

If yes, specify the risk factors ......…………………………………………

If no, give reasons …………........…………………………………………

3. Do you think it is safe to eat infected pork with cysticerci?

(1) Yes

(2) No

(3) Don’t know

4. What measures do you take when you find pork infected with cyst?

(1) Consult veterinary officer

(2) Condemn and not eat

(3) Thoroughly cooking and eat

(4) Sell pork to others

(5) Other measures

If other measures, specify which ………………………………………………….

**Practices**

1. Do you keep pig?

(1) Yes

(2) No

If yes, how many pigs do you have? ................................................................

2. How do you normally keep pigs during farming season? (more than one answer possible)

(1) Confinement

(2) Free roaming

(3) Tethering

(4) Others

If confinement, specify how ………………………………………………………

If others, specify the type …………………………………………………………

3. How do you normally keep pigs after crop harvesting? (more than one answer possible)

(1) Confinement

(2) Free roaming

(3) Tethering

(4) Others

If confinement, specify how ………………………………………………………

If others, specify the type …………………………………………………………

4. Do you think it is harmful if pigs roam freely?

(1) Yes

(2) No

(3) Don’t know

If yes, specify why …………………………………….

5. Have you ever slaughtered a pig at home?

(1) Yes

(2) No

If yes, was there a meat inspection?

(1) Yes

(2) No

If not inspected, what were the reasons? (more than one answer possible)

(1) Lack of transport for meat inspector

(2) High inspection fee that pig farmers can’t afford

(3) Shortage of meat inspectors/public health workers

(4) Poor infrastructures (difficult terrain)

(5) Vast/large areas for livestock field officer to cover

(6) No need for meat inspection

(7) Other reasons

If other reasons, specify which ……………………………………………………….

6. Do you eat pork?

(1) Yes

(2) No

If yes, can you identify ‘measly’ pork/white nodules/cysts? (show picture of pork infected with cysticerci)

(1) Yes

(2) No

7. Where do you normally eat pork? (more than one answer possible)

(1) At home only

(2) At neighbour’s house

(3) At village market

(4) Ate pork before, not eating anymore

If at home, how is your pork prepared before eating?

(1) Does not cook

(2) Well-cooking

(3) Well cooking and frying

(4) Moderate cooking

(5) Other methods

If other cooking methods, specify which …………………………………………

8. Do you wash vegetables and fruits to be eaten raw?

(1) Yes

(2) No

(3) Sometimes

9. Do you always use a toilet?

(1) Yes, always

(2) Sometimes

(3) No, never

If never, give reasons why not ……………………………………………

If sometimes, give reasons why only sometimes…………………………

10. Do you always wash your hands after using a toilet?

(1) Yes, always

(2) Sometimes

(3) No, never

If yes, do you use soap?

(1) Yes, always

(2) Sometimes

(3) No, never

11. Do you wash your hands before eating?

(1) Yes, always

(2) Sometimes

(3) No, never

If yes, do you use soap?

(1) Yes, always

(2) Sometimes

(3) No, never

12. How do you treat water before drinking? (more than one answer possible)

(1) Boil

(2) Filter

(3) No treatment

(4) Other methods

If other methods, specify which …………………………………………………
